# Phase-pure VO_2_ nanoporous structure for binder-free supercapacitor performances

**DOI:** 10.1038/s41598-019-40225-1

**Published:** 2019-03-15

**Authors:** Raktima Basu, Subrata Ghosh, Santanu Bera, A. Das, S. Dhara

**Affiliations:** 1 Surface and Nanoscience Division, Indira Gandhi Centre for Atomic Research, Homi Bhabha National Institute, Kalpakkam, 603102 India; 2 Water and Steam Chemistry Division, BARC Facility, Homi Bhabha National Institute, Kalpakkam, 603102 India

## Abstract

Vanadium oxides are anticipated as a high-performance energy storage electrode due to their coupled double layer and pseudo-capacitative charge storage mechanism. In the present work, we investigated the influence of different structural phases of as-grown VO_2_ nanoporous structure and corresponding oxidation states on the supercapacitor performance. This nanoporous structure facilitates fast ion diffusion and transport. It is shown that stoichiometric monoclinic VO_2_, with V oxidation state of +4, provides superior charge storage capacity with a capacitance value of 33 mF/cm^2^, capacitance retention of 93.7% and Coulombic efficiency of 98.2%, to those for VO_2_ structures with mixed oxidation states of V^5+^ and V^4+^. A comparable high energy density is also recorded for the sample with all V^4+^. Scanning Kelvin probe microscopy results clarify further the formation of space charge region between VO_2_ and carbon paper. These key findings indicate the potentiality of binder-free single phase monoclinic VO_2_ porous structure towards the next-generation micro-supercapacitor application.

## Introduction

Supercapacitors (SC) are considered as one of the emerging technologies for energy storage devices because of their longer cycle life, higher power density, and environmental friendliness compared to the conventional batteries^1–3^. However, a plethora of significant research hunt is directed either towards finding a suitable electrode material from the past decades or adopting novel strategy to meet the energy requirement. In particular, carbon materials, metal oxide or hydroxide and conducting polymer are choices of suitable candidates as electrodes for SC^4–6^. Carbon materials provide good conducting pathways to the electrolyte ion for electric double layer (EDL) formation, chemical stability, and excellent electrical conductivity^7^. However, the charge storage capacity is limited^4^. The conducting polymer is also an attractive choice as a pseudo-capacitor^6^, but the electrochemical stability of it is poor. In this context, transition metal oxides (TMOs) are fascinating candidates on account of their variable oxidation states and hence rapid redox kinetics^5^. There is an enormous impetus to improve the energy density for their commercialization. Despite the availability of other TMOs^8–10^, such as RuO_2_, MnO_2_, Fe_2_O_3_, ZnO, In_2_O_3_, vanadium oxides are attractive candidates as SC electrode owing to their catalytic nature as well as low-cost and abundant storage on earth^11–13^. The poor charge storage properties and electrical conductivity of V-based materials can be overcome by fabricating directly on the current collector. There are several reports on SC properties of VO_x_ composites^7,14–25^. Supercapacitor performance in V_2_O_5_ is reported to be superior in most of the reports^18,20–22^. It may be noted here that the most prominent reports^18,21^, claiming high supercapacitance property of VO_2_, are either V_2_O_5_ or a composite with it. However, in a seminal report by Shao *et al*.^22^, VO_2_ with possible mixed phases showed better performance as supercapacitor compared to that for the well-known V_2_O_5_. This is because of the higher electronic conductivity in VO_2_ arising from a mixed-valence and structural stability due to the increased edge sharing and the consequent resistance to lattice shearing during cycling as reported by Lampe-Onnerud *et al*.^26^. Issues related to the presence of multiple valence states of V^13^, as well as its stability affecting capacitance retention and efficiency is found to impede further utility in SCs. In this context, the role of varied structural forms of VO_2_, which is reported to be a better electrode than V_2_O_5_, is yet to be understood. In our previous report^27^, we have shown VO_2_ can be grown with different distinct phases of monoclinic, triclinic and their admixture, by controlling the carrier gas flow rates.

Vanadium oxides find enormous attraction because of their well-known metal to insulator transition (MIT) and multi-valency. V is a transition metal ([Ar]3*d*^3^4*s*^2^) having valences ranging between +2 to +5 with principal oxides in the form of VO, V_2_O_3_, VO_2_, and V_2_O_5_, respectively. However, the V-O phase diagram includes mixed valence oxides containing two oxidation states, such as V_6_O_13_, V_8_O_15_, V_7_O_13_, V_6_O_11_, and others allowing an easy conversion between oxides of different stoichiometry. VO_2_ is particularly interesting to study because of the fact that the MIT occurs at a technologically important temperature of 340 K^28^, which is close to room temperature (RT), along with a structural transition between low-temperature monoclinic (M1) to high-temperature rutile tetragonal (R) phase^29,30^ via two intermediate phases of monoclinic M2 and triclinic T (or monoclinic M3)^31^. The M2 and T phases can be stabilized at RT by local variation in the stoichiometry introducing native defects^27^. VO_2_ undergoes several structural phase transitions as a function of temperature, external electric field, hydrostatic pressure, intense illumination, and strain^27^. So, it is significant to find out the influence of each structural phase and corresponding oxidation states of VO_2_ on supercapacitive behaviour. The present study not only stresses the betterment of results with vanadium oxide but also attempts to provide insight into how different phases of VO_2_ contribute and behave electrochemically.

In this study, we report supercapacitive performance of different structural phases of VO_2_ nanoporous structures on carbon fiber for the first time, with no binder to fabricate the electrode. The different phases of VO_2_ from M1 to T are grown by controlling the O content in the chemical vapor deposition (CVD) technique. The as-grown phase of the monoclinic VO_2_ structure (M1) shows excellent SC performance with higher specific capacitance than those of the other two phases. The plausible formation of space charge capacitance is also discussed further to understand the supercapacitive performance. Further electrochemical impedance spectroscopy (EIS) is carried out to probe SC performance. Our aim is to explore and contribute to the gap area of the charge storage capacity and electrochemical stability in a supercapacitor with regard to VO_2_. Noteworthy, we provide clear explanation and relevance of oxidation state and work-function of the corresponding phases of VO_2_.

## Results and Discussions

### Morphological analysis

**Figure 1:**
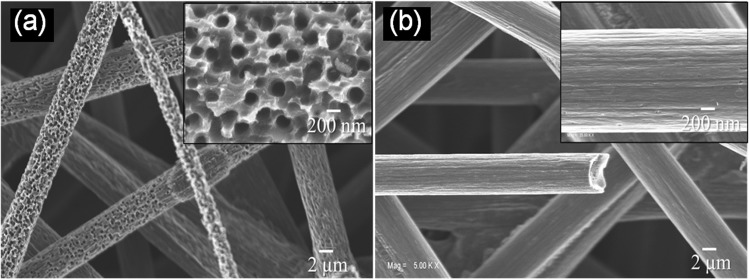
Scanning electron micrograph of (**a**) VO_2_ nanoporous structure grown on carbon paper and (**b**) bare carbon fiber. Insets show the magnified images with a typical pore diameter of approximately 200 nm.

To investigate the coating of VO_2_ structure on the carbon fiber, field-emission scanning electron microscopic (FESEM) analysis is performed, and typical micrograph is shown in Fig. 1. The conformal coating of VO_2_ porous structure on the carbon fiber is shown in Fig. 1a. The structure consists of nanopores with average pore diameters of approximately 200 nm, as observed from the inset of Fig. 1a. The as-grown samples S1, S2, and S3 show similar morphologies and pore diameter (Supplementary Fig. S1). The thickness of the VO_2_ nanoporous layer is found to be around 800(±50) nm for all the samples from the cross-sectional FESEM studies (Supplementary Fig. S2). To ensure its porous nature after the growth of VO_2_, FESEM images obtained from the bare carbon fiber is shown in Fig. 1b. Expectedly, V_2_O_5_ gets reduced to VO_2_ after reacting with C and releases CO_2_ in forming nanoporous structure^32^,

1 $${{\rm{2V}}}_{{\rm{2}}}{{\rm{O}}}_{{\rm{5}}}+{\rm{C}}\to {{\rm{4VO}}}_{{\rm{2}}}+{{\rm{CO}}}_{{\rm{2}}}$$

The ordered nanopores in the VO_2_ structure can offer large surface area and shortest channel/pathways for the electrolyte ions for enhanced SC performance.

### Structural analysis

**Figure 2:**
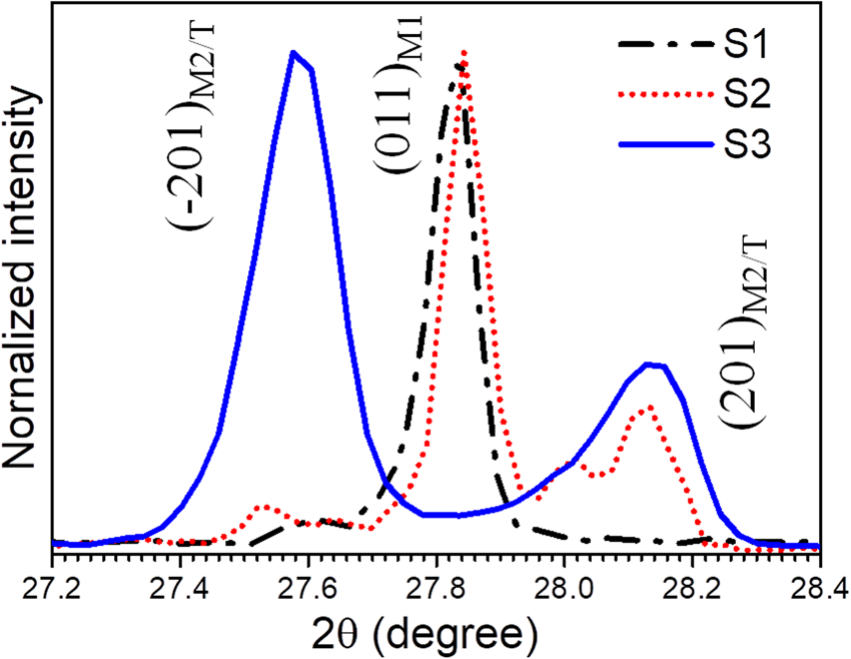
Glancing incidence X-ray diffraction spectra of the pristine samples S1, S2, and S3 indicating crystallographic (*hkl*) planes of the corresponding phases.

Crystallographic structural analyses are shown (Fig. 2) for samples grown with varying O content. The monoclinic M1 phase of VO_2_ is confirmed in sample S1 from the diffraction peak at 2θ = 27.83°, which is assigned as (011) plane (JCPDS # 04-007-1466). Whereas M2 (JCPDS # 00-033-1441) or T (JCPDS # 01-071-0289) phase of VO_2_ shows diffraction peaks and corresponding (*hkl*) planes at 27.58° (201) and 28.14° (2̄01) for the S3 sample. In case of sample S2, observation of diffraction peaks and corresponding crystalline planes at 2θ = 27.83° (011)_M1_, 27.58° (201)_M2/T_ and 28.14° (2̄01) _M2/T_ confirm the presence of both M1 and M2/T phases of VO_2_. The presence of M2 or T phase in the O rich samples (S2 and S3) suggests that V^4+^ state is replaced by V^5+^, as reported earlier by us^27^.

### Spectroscopic analysis

**Figure 3:**
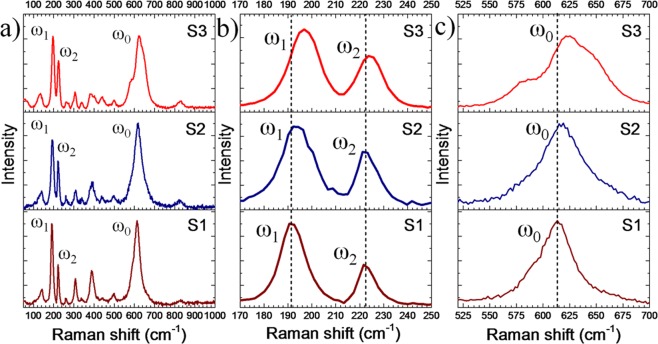
(**a**) Raman spectra of the pristine samples S1, S2 and S3 (**b**) Raman modes corresponding to V-V chain (ω_1_ and ω_2_) and (**c**) characteristic peak of VO_2_ (ω_0_) correspond to V-O stretching for the samples.

The mode of vibration and phase information of as-grown VO_2_ structures are investigated by Raman spectroscopy. The typical Raman spectra for the samples S1, S2, and S3 at RT are shown in Fig. 3. Based on the group theoretical analysis, predicted eighteen Raman-active phonon modes of VO_2_ at Γ point are for M1: 9*A*_g_ + 9*B*_g_; and for M2 and T: 10*A*_g_ + 8*B*_g_^33^. However, only eleven vibrational modes for sample S1 and S2 and twelve vibrational modes for sample S3 are observed (Fig. 3a). Raman modes at 141 (*A*_g_), 190 (*A*_g_), 221 (*A*_g_), 259 (either *A*_g_ or *B*_g_; *A*_g_/*B*_g_), 308 (*A*_g_), 339 (*A*_g_), 389 (*A*_g_/*B*_g_), 440 (*A*_g_/*B*_g_), 498 (*A*_g_/*B*_g_), 612 (*A*_g_), 829 (*B*_g_) cm^−1^ confirm the presence of pure M1 phase of VO_2_ in sample S_1_ and is in good agreement with earlier reports^34,35^. The Raman modes denoted as ω_0_, ω_1,_ and ω_2_ (Fig. 3) correspond to V-O vibrations, the vibration of V ions along the *c*-axis and in the transverse direction, respectively. The two major differences are observed in the Raman spectra of the samples; (i) blue shift of the Raman modes at 190 cm^−1^ (ω_1_), and 221 cm^−1^ (ω_2_) by an amount of 7 and 4 cm^−1^, respectively (Fig. 3b) and (ii) significant blue shift of the Raman mode for 612 cm^−1^ (ω_0_) and appearance of a new peak at 578 cm^−1^ in sample S_3_ (Fig. 3c), as a signature of presence of the T phase of VO_2_^33^. Raman signals arising from VO_2_ on C papers and C^−^ related peaks from carbon paper beneath of VO_2_ could easily be identified by adjusting the depth of focusing using the confocal microscopy. Confocal Raman spectra were collected to confirm the presence of pure VO_2_ phases on the surfaces of the structures (Supplementary Fig. S3).

### Chemical analysis

**Figure 4:**
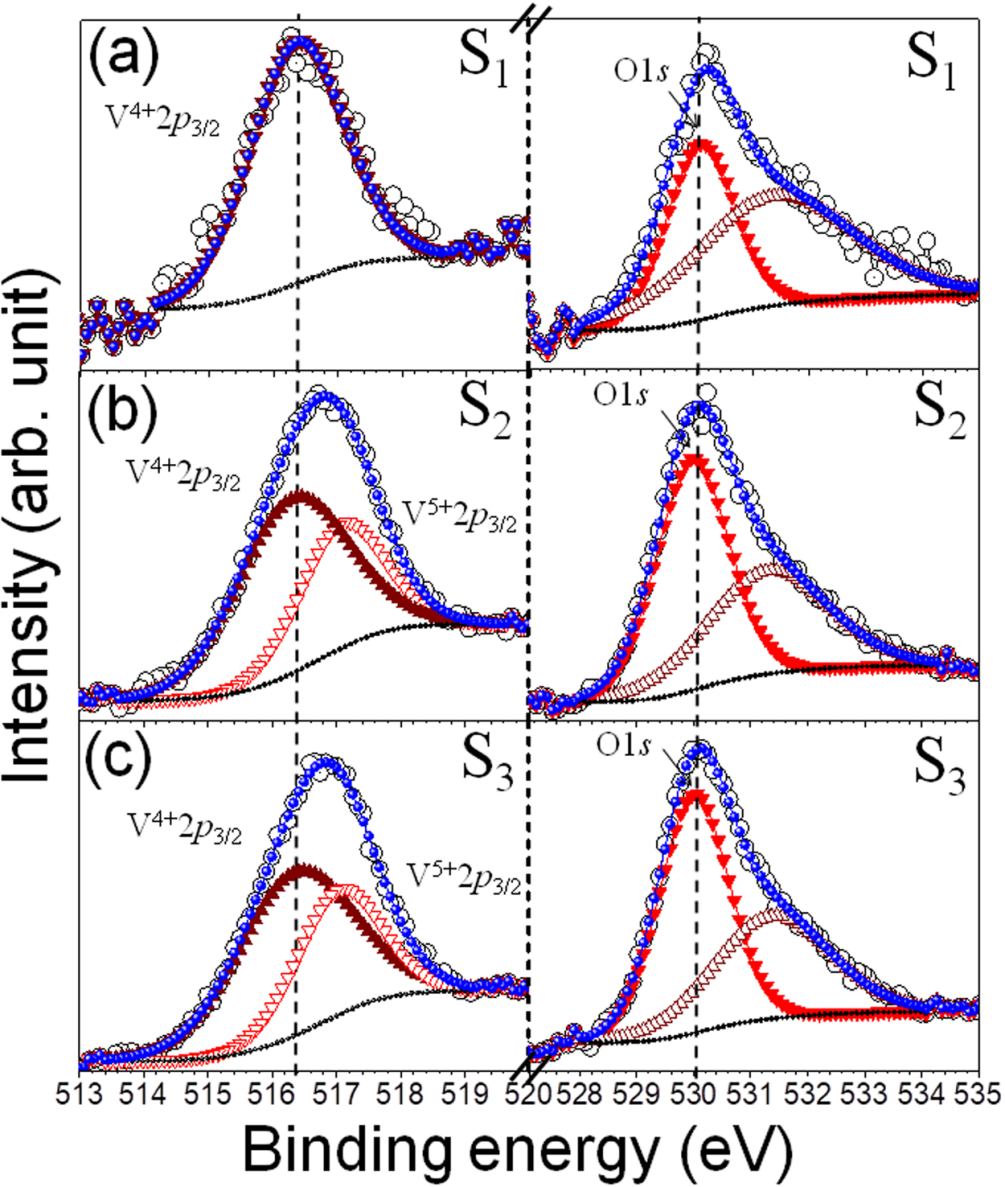
High resolution XPS spectra of the samples (**a**) S1, (**b**) S2, and (**c**) S3. Open circles represent the data points, solid arrows and circles represent the fitted curves. In the left panel, solid and empty arrows correspond to V^4+^ and V^5+^ oxidization states, respectively. In the right panel, solid arrows correspond to lattice oxygen, and empty arrows correspond to oxygen from absorbed oxygen species. The vertical dashed lines denote the corresponding BE values for V^4+^ 2*p*_3/2_ and lattice O1*s* states.

In order to determine the chemical oxidation states of V and O, X-ray photo-electron spectroscopic (XPS) study was employed on the VO_2_ structures. The typical XPS spectra of V2*p*_3/2_ and O1*s* peak of VO_2_ are shown in Fig. 4a,b. Details of XPS result for these samples grown on Si can be found in our previous report, and we have shown that substrate-induced strain effect plays no role in the XPS result^27^. Presence of 2*p*_3/2_ peak at 516.3 eV is assigned as a V^4+^ state, and only V^4+^ state is observed for sample S1. The peak width (FWHM) of V 2*p*_3/2_ from sample S1 is found out as 1.7 eV which is increased to 2.0 and 2.1 eV in samples S2 and S3, respectively. The higher peak width is attributed to the presence of additional chemical state in the samples. Hence, the peaks in sample S2 and S3 are deconvoluted, and a suitable peak fitting gives rise to a peak at 517.1 eV in addition to the peak at 516.3 eV. The peak evolved at 517.1 eV is assigned as V^5+^ state in samples S2 to S3 as proposed in the X-ray diffraction (XRD) study. The ratio of the area under the curves of the two peaks, i.e., V^5+^/V^4+^ increases progressively from S1 to S3. On the other hand, O1*s* peak at low BE value of 530 eV is attributed to O in the lattice, and that for high BE value at 531.5 eV corresponds to absorbed O species. The intensity and area under the curve of O1s are found to increase from sample S1 to S3 (Fig. 4b).

### Work function and surface charge analysis

The metastable T phase evolves with an increase in temperature during the transition from monoclinic M1 to rutile R phase^36^. The zeta potential of the three samples are found out to be −35.6, −30.3, and −14.4 mV for samples S1, S2, and S3, respectively. The decrease in the value of zeta potential with an increase in the V^5+^/V^4+^ ratio is in good agreement with the reported data^37^. The work function of the three samples are calculated from scanning Kelvin probe microscopic (SKPM) measurements as 5.35, 4.81, and 4.8 eV for samples S1 (M1), S2, and S3 (T), respectively (Supplementary Fig. S4). The work function of VO_2_ is reported to decrease with increasing temperature and expected to be less in T phase, than that of the M1 phase^38^.

### Supercapacitor performance

Investigation for the SC performance of the as-grown VO_2_/Na_2_SO_4_ system is carried out in the three-electrode electrochemical cell. The specific capacitance is calculated using the given formula:

2 $$\,{\boldsymbol{C}}=(\int {\boldsymbol{I}}{\boldsymbol{.dV}})/{\boldsymbol{v}}{\boldsymbol{.A}}{\boldsymbol{.V}}\,{\rm{from}}\,{\rm{cyclic}}\,{\rm{voltammogram}}\,(\mathrm{CV})$$

3 $${\boldsymbol{C}}=({\boldsymbol{I}}{{\boldsymbol{t}}}_{{\boldsymbol{d}}})/{\boldsymbol{V}}\,\,\,{\rm{from}}\,{\rm{charge}} \mbox{-} {\rm{discharge}}$$

where *I* is the charge-discharge current, *v* is scan rate; *A* is exposed area of the electrode in the electrolyte, ${{\boldsymbol{t}}}_{{\boldsymbol{d}}}$ is discharge time, ${\boldsymbol{V}}$ is the potential window.

**Figure 5:**
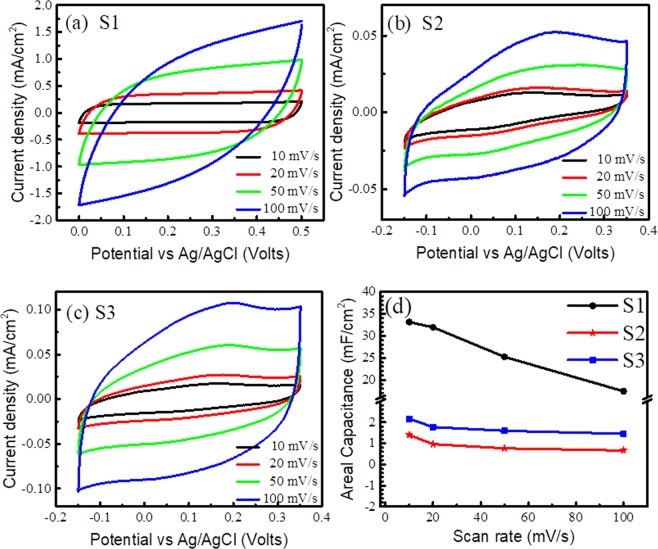
Cyclic voltammogram of (**a**) S1, (**b**) S2 and (**c**) S3. (**d**) Areal capacitance versus scan rate of VO_2_/Na_2_SO_4_ system.

The cyclic voltammogram (CV) plots of the studied samples are carried out from 10 to 100 mV/s at a potential window of 0.5 V *versus* Ag/AgCl (Fig. 5a–c). The near-rectangular CV with symmetrical shape is observed for the sample S1 at scan rates of 10 and 20 mV/s (Fig. 5a). The current density of the system is found to increase with increasing scan rate, suggesting fast kinetics and reversibility of the system. The absence of redox peak of S1 is attributed to mostly non-Faradic active sites for ion diffusion^39^. Hence, the obtained result ensures the fast electron transfer which is facilitated by VO_2_ nanoporous structure on the carbon fiber. The possible reactions are (i) surface adsorption/desorption of electrolyte cations on the vanadium oxide (Equation 4) and (ii) intercalation/deintercalation of electrolyte cations in the bulk vanadium oxide (Equation 5):

4 $${({{\rm{VO}}}_{{\rm{2}}})}_{{\rm{surface}}}+{{\rm{Na}}}^{+}+{{\rm{e}}}^{-}\leftrightarrow {({{{\rm{VO}}}_{{\rm{2}}}}^{-}{{\rm{Na}}}^{+})}_{{\rm{surface}}}$$

5 $${{\rm{VO}}}_{{\rm{2}}}+{{\rm{Na}}}^{+}+{{\rm{e}}}^{-}\,\leftrightarrow {{\rm{VO}}}_{{\rm{2}}}{\rm{Na}}$$

It is noteworthy that unlike sample S1, the signature of oxidation peak is observed for both the samples S2 and S3 (Fig. 5b,c). The shape of CV plots for both the cases is unaltered with respect to the scan rate confirming its reversible nature. Redox reactions in sample S2 and S3 can be initiated due to the presence of mixed valence states V^4+^ and V^5+^ in those samples. The areal capacitances of the samples are calculated using Equation 2. The estimated areal capacitance of samples S1, S2 and S3 are 33.15 ± 0.80, 1.39 ± 0.06 and 2.16 ± 0.04 mF/cm^2^, respectively. The minor variations in the capacitance values represent the statistics gathered from the measurement on more than one sample of each type. The CV plot of the bare carbon paper was also carried out at the same potential window of 0.5 V *versus* Ag/AgCl, and the areal capacitance was found out to be 85 μF/cm^2^ (not shown in figure). The value is significantly smaller than those for any of the VO_2_ coated samples showing that the major contribution of areal capacitance is essentially from VO_2_. According to Equation 1, there was a loss of CO_2_ from the substrate where the samples were synthesized by the CVD technique at 1150 K for 3 hrs. The loss of CO_2_ affects the weight of C paper. In that case, using the weight of carbon paper as a reference becomes ambiguous for measuring the weight of VO_2_. So, following other references where the geometrical surface is taken for quantification^20,40–44^, the capacitance value is described in F/cm^2^. Moreover, the surface area for all the samples is approximately the same as shown from FESEM studies (Supplementary Fig. S1). Sample S1 shows a higher capacitance value than the reported values of other materials and the comparison is listed in Table 1. Although capacitance values of S2 and S3 samples in T phase are higher than those of Al_2_O_3_-TiO_2_, multi-layer TiO_2_, carbon nanotube and vertical graphene nanosheets, however, they are one order lower than that of sample S1 (M1 phase). The observed result, which matches well with the existing report by Tang *et al*.^19^, is attributed to the O content of VO_2_ structure. The quasi-rectangular shape of CV plots and decreased capacitance at higher scan rate (Fig. 5d) are attributed to the inaccessibility of electrolyte ion interior of the active material. The higher capacitance value for sample S1 can be understood from the double layer formed at the interface. The work function of sample S1 is found to be 5.35 eV, whereas, in the case of sample S2 and S3, the work function values are ~4.8 eV. As the work function^45^ of carbon (C) is 4.81 eV, it is quite expected that at the interface of VO_2_/C, there is a probability of formation of space charge region for sample S1 only. EDL at S1/C interface contributes to the space charge capacitance for sample S1.

**Table 1:** Comparison of supercapacitive performance of VO_2_ structures with other nanostructures.

Sample	Areal Capacitance mF/cm^2^	References
Multi-layer TiO_2_ nanotube	0.06	Zheng *et al*.^40^
Carbon modified multi-layer TiO_2_ nanotube	3.75	
Vertical graphene	0.56	Ghosh *et al*.^41^
MnO_2_-Vertical graphene	5.64	
Al_2_O_3_-TiO_2_	0.13	Du *et al*.^42^
Carbon nanotube	0.46	Zhou *et al*.^43^
Carbon nanotube/Polypyrrole	18.25	
TiVN	15	Achour *et al*.^44^
Monoclinic (M1) VO_2_	33.15	This work

**Figure 6:**
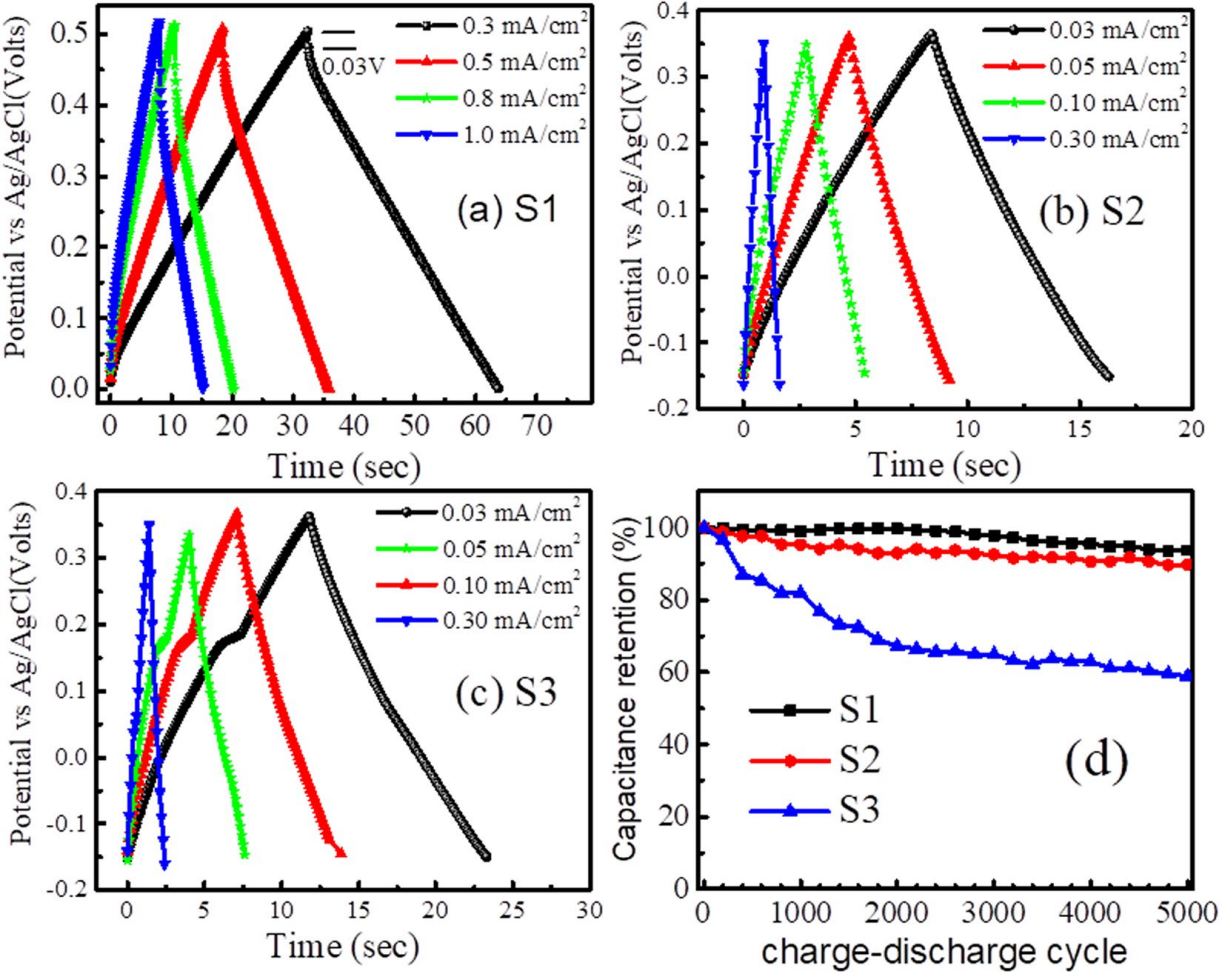
Charge-discharge profile at current density of 0.3 to 1.0 mA/cm^2^ (**a**) S1, (**b)** S2 and (**c**) S3 (**d**) capacitance retention of the samples with charge-discharge cycle.

The SC behavior of VO_2_ nanoporous structures is also confirmed from the charge-discharge profile at different current density ranges from 0.3 to 1.0 mA/cm^2^ (Fig. 6a–c). Excellent SC behavior includes a linear and symmetric charge-discharge profile for samples S1 and S2, as shown respectively in Fig. 6a,b. The insignificant potential drop of 0.03 V in the discharge profile of sample S1 is attributed to the charge transfer resistance and electrode-electrolyte resistance (Fig. 6a). At a current density of 0.3 mA/cm^2^, the specific capacitance of sample S1 is found to be 20.7 mF/cm^2^. The reduced specific capacitance of 14.7 mF/cm^2^ is observed at a higher current density of 1 mA/cm^2^. However, a non-linear charging-discharging profile is observed for the sample S3, implying the poor surface charge capability in the sample, which may be correlated to the low value of zeta potential for this sample. Sample S3 is in the T phase of VO_2_ containing the maximum amount of V^5+^ (*d*^0^) states (holes), and it can resort to the oxidation process easily to maintain charge neutrality. Obtained capacitance values of samples S2 and S3 are 0.47 and 0.68 mF/cm^2^, respectively. Low capacitance values of samples S2 and S3, as compared to that for sample S1, matches with CV results where one order higher value of areal capacitance is calculated for sample S1.

The cyclic stability is one of the key parameters for high-performance SC. Coulombic efficiency of the samples is obtained from the equation,

6 $${\boldsymbol{\eta }}={{\boldsymbol{t}}}_{{\boldsymbol{d}}}/{{\boldsymbol{t}}}_{{\boldsymbol{c}}}\,$$

where *t*_*c*_ is the charge time.

The capacitance retention of 93.7% with a Coulombic efficiency of 98.2% for 5000 charge-discharge cycles suggests excellent rate capability, reversibility, and durability of sample S1 (Fig. 6d). The cyclic stability is found to be superior to recently studied VO_2_(B)/C core-shell by Zhang *et al*.^46^. Whereas, sample S2 shows capacitance retention of 89.8% with a Coulombic efficiency of 98.5% and sample S3 shows the capacitance retention of 58.9% with a Coulombic efficiency of 95.6% (Fig. 6d). The low value of capacitance retention in case of S3 is because of poor surface charge potential as observed in charge-discharge profile. All the samples were re-examined by Raman spectroscopy and FESEM studies and found out to be stable after several runs.

**Figure 7:**
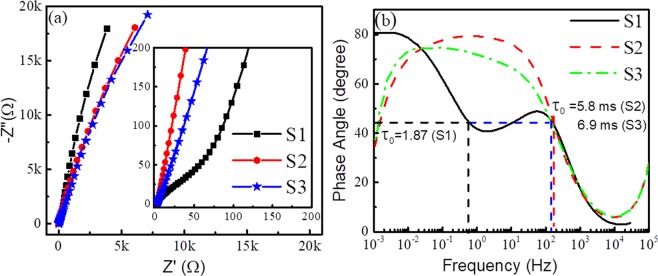
(**a**) Nyquist plot and (**b**) Bode plot of VO_2_/Na_2_SO_4_ system.

To probe frequency behavior of the VO_2_/Na_2_SO_4_ system, EIS analysis is carried out, and the extracted result in terms of Nyquist and Bode plots are shown in Fig. 7. The steeper vertical line of sample S1 is observed form the Nyquist plot, as compared to those for samples S2 and S3. The results further support the superior SC behavior of sample S1, as compared to those for other two samples. The intercept in real impedance axis is known as equivalent series resistance (R_s_) which includes resistances contributed from solution, electrodes and electrode/electrolyte. The R_s_ is found to be 2.81 Ω for samples S2 and S3 and 46 Ω for sample S1 (Fig. 7a), representing good electrical contact between VO_2_ and carbon paper. The semi-circular arc represents the double layer formation^47^ between active material and electrolyte. This signature is observed from the shape of CV plot (Fig. 5). However, a semicircular arc at high frequency is observed only for sample S1 from the Nyquist plot, as depicted in the inset of Fig. 7a. The disappearance of semicircular arc in samples S2 and S3 can be explained by the space charge polarization at the interface between VO_2_ and carbon paper, as expected from SKPM results which indicate similar work function for these samples and carbon.

The phase angle with respect to the frequency (Bode plot) is plotted in Fig. 7b. The phase angle of 81° at lower frequency region intuitively reveals the better-quality supercapacitive behavior in sample S1, as it approaches a phase angle of an ideal SC of 90°. Whereas, stability issue is observed for samples S2 and S3, which in-turn show reduced phase angle at a lower frequency. These results support our charge-discharge data (Fig. 6d). The Bode plot comprises a hump around 100 Hz for the sample S1 (Fig. 7b) confirming the excellent electrolyte ion accessibility and capacitance retention^48^. Moreover, the time constant of the system demarcating the power delivering capability is obtained from the Bode plot at a phase angle of 45°. Two-time constants are estimated for the sample S1 are 1.87 s and 5.8 ms, which again prove the contribution from space charge polarization at S1/C interface. Time constants for samples S2 and S3 are found to be 5.8 and 6.9 ms. The low time constants suggest the fast charge deliver capability of the VO_2_/Na_2_SO_4_ system.

The specific (sp.) energy densities (E) and power densities (P) of the samples are calculated using Equations (7,8).

7 $${\bf{E}}={\bf{C}}{{\bf{V}}}^{2}/2$$

8 $${\rm{and}}\,{\bf{P}}={\bf{E}}/{{\bf{t}}}_{{\bf{d}}}$$

**Figure 8:**
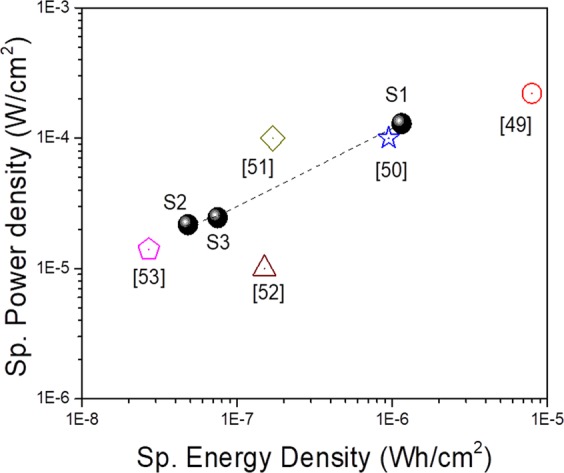
A Ragone plot of sp. energy density versus sp. power density for VO_2_ nanoporous structures. The data is compared with the published performance of other supercapacitors. Filled balls represent the data points of this work for sample S1, S2, and S3. The dashed line is guide to the eye. The symbols represent the data from the literature with the corresponding references below.

The sp. power densities for sample S1, S2, and S3 are found out as 129.3 ± .80, 21.7 ± 0.6, and 24.5 ± 0.4 μW/cm^2^, respectively. The dependence of areal energy density on areal power density is shown in the Ragone plot (Fig. 8).

The maximum measured energy density of the presently investigated supercapacitor is 1.15 ± 0.80 μWh/cm^2^, which is of the same order to that for the flexible supercapacitors comprising hierarchical nanostructures with carbon spheres and graphene oxide nanosheets (7.96 μWh/cm^2^)^49^. However, the value is one order higher than Yarn supercapacitor based on PANI/stainless steel (0.95 μWh/cm^2^)^50^, graphene fiber supercapacitor (0.17 μWh/cm^2^)^51^, and Yarn supercapacitor based on CNT and Ti fibres (0.15 μWh/cm^2^)^52^. Though a high energy density and power density (one order higher than the data presented in this study) are reported for VO_x_ nanostructures on 3D graphene^20^, and Polyaniline (PANI)^21^ the data are not for phase-pure VO_2_. The phase of the vanadium oxide presented by those groups resembles V_2_O_5_ rather than VO_2_ as understood from the XRD and Raman studies reported by them. The energy densities for sample S2 and S3 are 48 ± 0.06 nWh/cm^2^ and 75 ± 0.4 nWh/cm^2^, respectively, which is two orders less compare to sample S1 but higher than ZnO nanowire/MnO_2_ fiber supercapacitor (27 nWh/cm^2^)^53^.

## Conclusion

We have shown the potential ability of different structural phases and corresponding oxidation states of VO_2_ nanoporous structure for supercapacitor (SC) properties for the first time. Monoclinic VO_2_ sample with all V in oxidation state +4 shows better SC performance with the capacitance of 33 mF/cm^2^, Coulombic efficiency of 98.2% and 93.7% capacitance retention after 5000 cycles, as compared to those for VO_2_ samples with triclinic and mixed phases. A comparable high energy density is recorded for the sample with all V^4+^ in the Ragone plot. The power densities of the samples also show their mutual dependence indicating comparable values with the performance of other supercapacitors. The space charge region, formed at the interface of VO_2_ and carbon paper, is found to contribute in the observed superior capacitance value for the pure M1 phase of VO_2_. As VO_2_ undergoes several structural phase transitions with a small variation in temperature, external electric field, hydrostatic pressure, intense illumination, and strain, our study provides insight on how different phases of VO_2_ contribute and behave electrochemically.

## Methods

### Materials detail

Analytical grade pure V_2_O_5_ powder (Sigma-Aldrich, 99%) and sodium sulphate (Na_2_SO_4_) (Merck Life Science Pvt. Ltd., India) were utilized without any further chemical purification. Milli-Q water with a resistivity of 18 MΩ cm was utilized in the experiments.

### Growth of VO_2_

**Figure 9:**
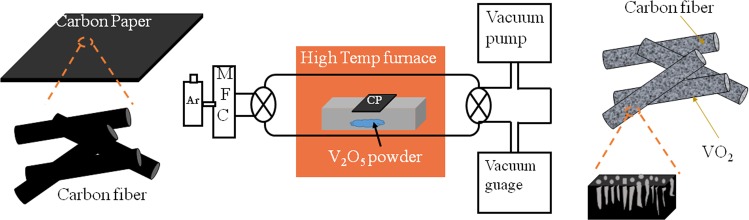
Schematic of nanoporous VO_2_ fabrication.

VO_2_ samples were grown by vapor transport process using bulk V_2_O_5_ powder as source and Ar (99.9%) as a carrier gas. Carbon paper, composed of an open mesh of carbon fibers was used as a substrate and placed on the high pure (99.99%) alumina boat inside a quartz tube. The source was placed in an alumina boat, which was kept in a furnace and was pre-evacuated up to 10^−3^ mbar. The substrate was kept above the source and downstream to the Ar flow direction. The samples were prepared with Ar flow of 10, 20 and 30 sccm named as S1, S2, and S3, respectively. The synthesis was carried out for 3 hrs at an optimized growth temperature of 1150 K. The schematic of growth is depicted in Fig. 9. As-grown VO_2_ structures on carbon paper were used as binder-free SC electrode. Noteworthy to mention that fabrication of the electrode did not require any additional steps such as preparing a slurry by mixing active material, binder and conductive additive in a solvent, coating slurry on a current collector and drying at a certain temperature for several hours.

### Morphology, phase, and structural characterization techniques

The surface morphology of the grown VO_2_ structures on carbon paper was imaged by a field-emission scanning electron microscope (Supra 55, Carl Zeiss, Germany). The crystallographic structural analysis and phase confirmation of VO_2_ were studied by glancing incidence X-ray diffractometer (Inel, Eqinox 2000) with a glancing angle (θ) of 0.5° in the θ − 2θ mode and Cu K_α_ was used as the radiation source (λ = 1.5406 Å). A Raman spectrometer (in-Via, Renishaw, UK) with a monochromatic and coherent excitation of 514.5 nm Ar^+^ laser source was used to analyze the vibrational properties of the grown sample in the backscattering configuration. An 1800 grooves/mm grating was used as a monochromator, and thermoelectrically cooled CCD camera was utilized as a detector for the scattered waves. The X-ray photoelectron spectroscopy analysis was performed using VG ESCALAB MK200X spectrometer for the VO_2_ nanoporous samples synthesized at different growth conditions. An X-ray source of Al-Kα (1486.6 eV) was used with beam diameter around 3 mm and collection area (with the largest slit) approximately 2 × 3 mm^2^. The pass energy of the hemispherical analyzer was kept 20 eV with an expected spectral resolution of 0.4 eV for high resolution data collection. The binding energy (BE) values were measured with respect to the C 1 *s* reference peak, and the spectra were processed by applying Shirley type background. The curves were fitted by a mixture of Gaussian–Lorentzian line shapes.

### Electronic and electrochemical characterization

The stability in aqueous solution and surface charge information of the samples were studied by the zeta potential (Malvern’s Zetasizer-Nano) measurements. To understand the formation of space charge region, work functions of the samples were found out by *in situ* scanning Kelvin probe microscopy. The contact potential difference (CPD) between the samples and the electrically conductive tip (SCM-PIT; Pt-Ir coated Si tip) was measured at RT using an Agilent 5500 with a two-lock-in amplifier setup. The measurements were performed at ultra-high vacuum (~10^−7^ mbar) with a VAC bias at frequencies in the range 10–15 kHz plus a DC bias between the sample and an electrically conductive tip (SCM-PIT; PtIr coated Si tip). The CPD can be written as

9 $${{\rm{V}}}_{{\rm{CPD}}}=({{\rm{\varphi }}}_{{\rm{M}}}-{{\rm{\varphi }}}_{{\rm{S}}})/e$$

where ϕ_M_ and ϕ_S_ are work function of the metal tip and the sample, respectively, and *e* is the elementary charge. Any change in the work function of the sample is reflected directly in the CPD values.

Supercapacitive performances of the VO_2_ samples including cyclic voltammetry, charge-discharge profile and EIS were investigated by three-electrode electrochemical work station (Metrohm-Autolab, model PGSTAT302N). Ag/AgCl (3 M KCl saturated) and Pt foil (1 × 1 cm^2^) were used as a reference and counter electrodes, respectively. A (1 × 2 cm^2^) carbon paper was used as a substrate for growth of all the samples, where (1 × 1 cm^2^) area was masked to avoid any VO_2_ deposition. The rest (1 × 1 cm^2^) area, completely coated with VO_2_, was used as the working electrode. All the three electrodes were mounted by lids and immersed vertically down in 1 M Na_2_SO_4_ electrolyte media. The cell is assembled such that only the VO_2_ coated area is exposed to the electrolyte and the electrical contact area is away from the electrolyte level. The cyclic voltammetry behaviors at scan rates ranging from 10 to 100 mV/s and charge-discharge cycles at different current densities from 0.3 to 1.0 mA/cm^2^ were carried out in a potential window of 0.5 V. EIS was performed in the frequency range 10 kHz to 0.001 Hz with open circuit potential, with an alternative current perturbation of 10 mV. The Nyquist plots were drawn from the impedance data. Before, SC investigation, all samples were presoaked in the electrolyte for better wettability.

## Supplementary information

Phase-pure VO<sub>2</sub> nanoroporous structure for binder-free supercapacitor performances
