# Supplementary material for: Phase-pure VO2 nanoporous structure for binder-free supercapacitor performances
Source: Sci Rep. 2019 Mar 15;9:4621. doi: 10.1038/s41598-019-40225-1 (PMC6420617; doi:10.1038/s41598-019-40225-1)
Supplement: Supplementary file 1 — Phase-pure VO2 nanoroporous structure for binder-free supercapacitor performances [file 41598_2019_40225_MOESM1_ESM.docx]

*Supplementary information*

**Phase-pure VO_2_ nanoporous structure for binder-free supercapacitor performances**

Raktima Basu,^*1^ Subrata Ghosh,^*1^ Santanu Bera,^2^ A. Das,^1^ S. Dhara,^*1^

^1^Surface and Nanoscience Division, Indira Gandhi Centre for Atomic Research, Homi Bhabha National Institute, Kalpakkam-603102, India

^2^Water and Steam Chemistry Division, BARC Facility, Homi Bhabha National Institute, Kalpakkam-603102, India


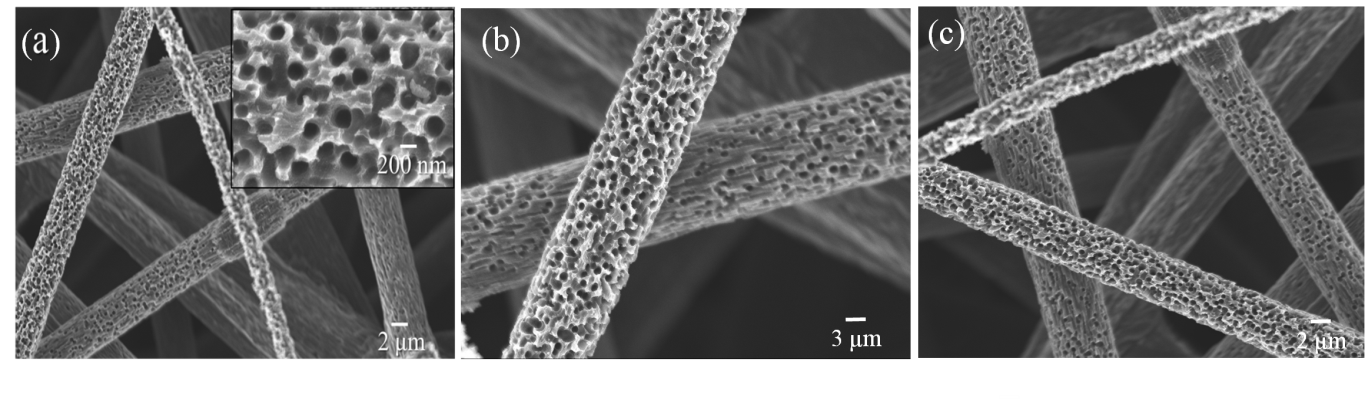


**Fig. S1** FESEM images of sample (a) S1, (b) S2, and (c) S3. Inset of (a) shows the magnified image with a typical pore diameter of approximately 200 nm.

**Fig. S2** Cross-sectional FESEM images of the samples (a) S1, (b) S2, and (c) S3 showing the thickness of VO_2_ layer around 800(+50) nm. (d) Typical high magnification image for the sample S3 shows the layer thickness of VO_2_ as approximately 800 nm.


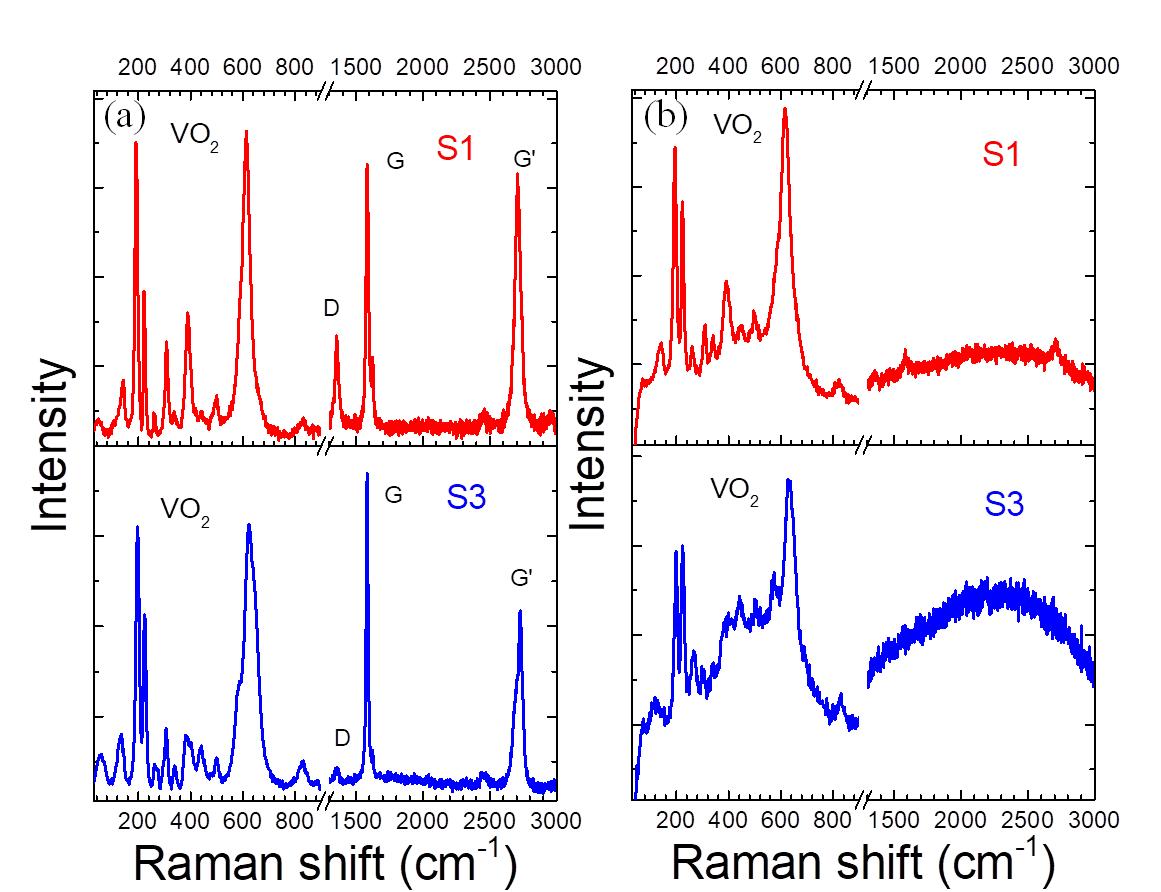


**Fig. S3** (a) Normal and (b) confocal Raman spectra of the pristine samples S1 and S3. The Raman modes below 1000 cm^-1^ correspond to VO_2_ and above 1000 cm^-1^ are due to carbon paper. Absence of Raman modes of carbon paper in confocal Raman spectroscopy confirms the surface of the material is constituted of pure VO_2_.


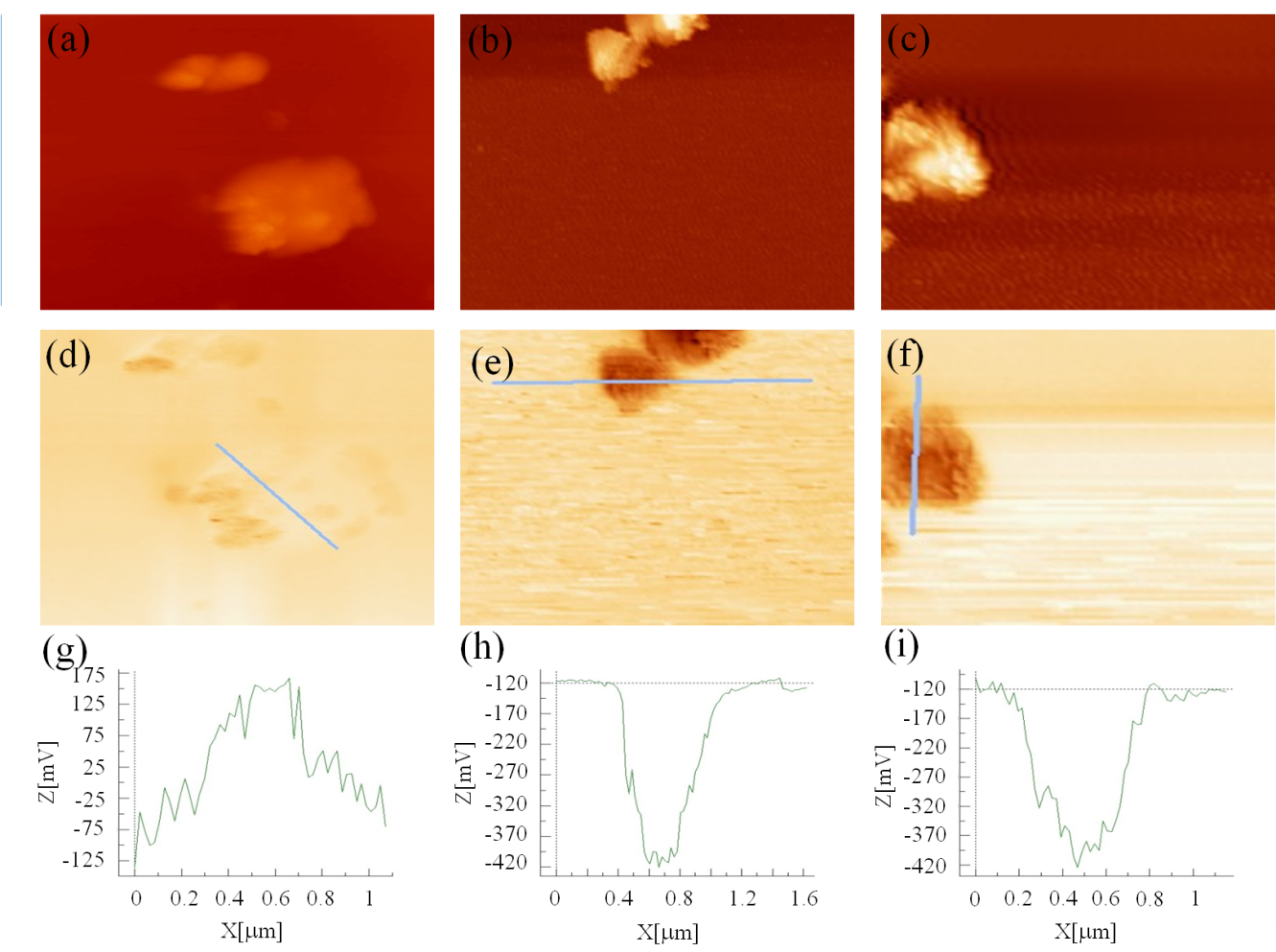


**Fig. S4** Topography of samples (a) S1, (b) S2, and (c) S3 with (d)-(f) and (g)-(h)showing corresponding SKPM images and the contact potential difference across the lines, respectively

The work functions of the samples are calculated from Scanning Kelvin probe microscopy (SKPM) measurements using 20 nm conductive PtIr coated Si tip. The work function of the tip was calibrated using Au reference and found out as 5.22 eV. Each line scan of SKPM mode is composed of a topography scan and a subsequent CPD measurement (Fig. S2). The φ values for the three samples found out as 5.35 eV, 4.81 eV, and 4.8 eV for sample S1, S2, and S3, respectively, using the equation:

V_CPD_ = (φ_M_ − φ_S_) / *e* …………....………………………. (1)

where φ_M_ and φ_S_ are work function of the metal tip and the sample, respectively, and *e* is the elementary charge.

**Growth of materials**:

Chemical vapor deposition (CVD) is a commercial process for depositing high-quality and high-performance materials. Due to its non-line-of-sight deposition capabilities, low cost, and high throughput, CVD finds its greatest application within the electronics industry. The first deposition technique, used for the deposition of VO_2_ thin films, was CVD (by Koide and Takei in 1967) and after half-a-century, it is still used extensively. It is well known that by varying experimental conditions, *e.g.*, substrate material, temperature, deposition time, and flow rate of reactor or carrier gas and others, materials with a wide range of physical, tribological, and chemical properties can be grown (Handbook of Deposition Technologies for Films and Coatings; Third Edition-chapter 7, 2010, 314-363). In the present work, the VO_2_ samples were grown on the same substrate (carbon paper) by vapor transport process using bulk V_2_O_5_ powder as source and Ar (99.9%) as a carrier gas. As the melting point of bulk V_2_O_5_ is 963K, we have varied the growth temperature starting from 1000 to 1200K in a step of 200K to know the optimum conditions to grow phase pure VO_2_. The growth time also was varied from 1 to 5 h to have a uniform coating of VO_2_ material. Finally, the optimized growth temperature and time for the synthesis of VO_2_ were found out as 1150K and 3 h, respectively. While keeping all the growth parameters identical, only the amount of gas exposure was varied (Ar flow of 10, 20 and 30 sccm for samples S1, S2 and S3, respectively). Since, the samples were grown at high temperature (1150K) with different percentage of flow of Ar (containing ≈2×10^5^ ppm O); a departure from perfect stoichiometry is probable, because of different amounts of O present in the carrier gas. The nonstoichiometry in VO_2_ introduced strain in the system and thereby stabilized the various metastable phases of VO_2_.
